# Osteoinductive Material to Fine-Tune Paracrine Crosstalk of Mesenchymal Stem Cells With Endothelial Cells and Osteoblasts

**DOI:** 10.3389/fbioe.2019.00256

**Published:** 2019-10-09

**Authors:** Hassan Rammal, Laura Entz, Marie Dubus, Aurélie Moniot, Nicolae B. Bercu, Johan Sergheraert, Sophie C. Gangloff, Cédric Mauprivez, Halima Kerdjoudj

**Affiliations:** ^1^EA 4691, Biomatériaux et Inflammation en Site Osseux (BIOS), SFR CAP Santé (FED4231), Université de Reims Champagne Ardenne, Reims, France; ^2^UFR d'Odontologie, Université de Reims Champagne Ardenne, Reims, France; ^3^EA 4682, Laboratoire de Recherche en Nanoscience (LRN), Université de Reims Champagne-Ardenne, Reims, France; ^4^Pôle Médecine bucco-dentaire, Hôpital Maison Blanche, Centre Hospitalier Universitaire de Reims, Reims, France; ^5^UFR de Pharmacie, Université de Reims Champagne Ardenne, Reims, France

**Keywords:** mesenchymal stem cells, paracrine activities, cell crosstalk, osteoinductive material, culture media

## Abstract

While stem cell/biomaterial studies provide solid evidences that biomaterial intrinsic cues deeply affect cell fate, current strategies tend to neglect their effects on mesenchymal stem cells (MSCs) secretory activities and resulting cell-crosstalks. The present study aims to investigate the impact of bone-mimetic material (B-MM), with intrinsic osteoinductive property, on MSCs mediator secretions; and to explore underlying effects on cells involved in bone regeneration. Human MSCs were cultured, on B-MM, made from inorganic calcium phosphate supplemented with chitosan and hyaluronic acid biopolymers. Collected MSCs culture media were assessed for mediators release quantification and used further to stimulate endothelial cells (ECs) and alveolar bone derived osteoblasts (OBs). Without osteogenic supplements, MSCs committed into bone lineage forming thus 3D bone-*like* nodules after 21 days. Despite a weak percentage of cell commitment, our data elucidate new aspects of osteoinductive material effect on MSCs functions through the regulation of the secretion of mediators involved in bone regeneration and subsequently the MSCs/ECs indirect crosstalk with osteogenesis-boosting effect. Using MSCs culture media, we demonstrate a large potential of osteoinductive materials and MSCs in bone regenerative medicine. Such strategies could help to address some insights in cell-free therapies using MSCs derived media.

**Graphical Abstract:**
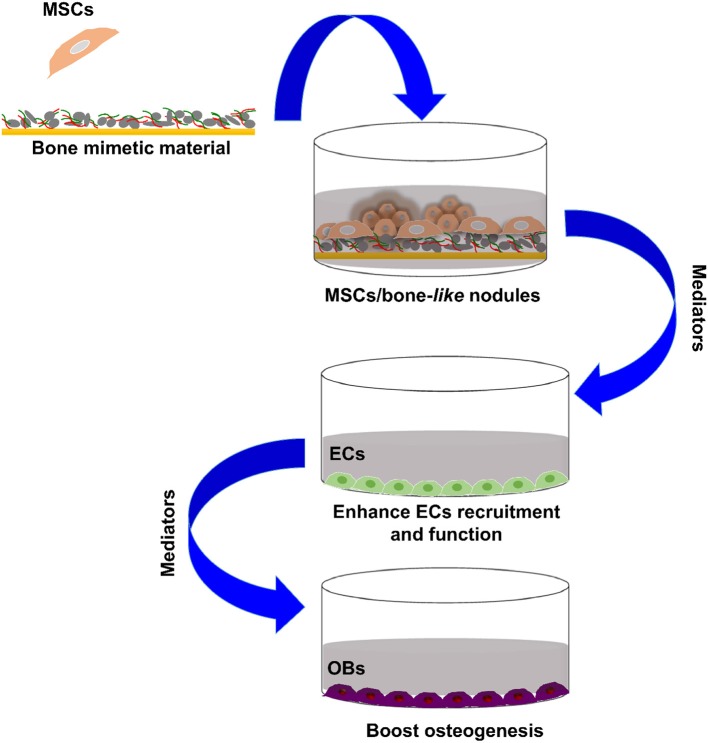
Bone forming cells paracrine crosstalk.

## Introduction

During the last decade, mesenchymal stem cells (MSCs) have been proven effective for bone regeneration as evidenced through *in vitro, in vivo* animal experiments and clinical trials (Asatrian et al., 2015; Jin and Lee, 2018). Scientific investigations have tried to understand the MSCs biological mechanism of action in skeletal tissue repair and to decipher their potential in cell therapy and regenerative medicine. Early MSCs studies in bone regenerative medicine were focused on their great potential to differentiate into multiple tissue types and supported the idea that MSCs have the capacity for tri-lineage differentiation into, osteoblasts (OBs), chondrocytes or adipocytes. Such optimism suggested that upon implanting, MSCs would colonize and differentiate at the bone lesion site along the osteoblastic lineage and thus replace damaged resident OBs (Bruder et al., 1994, 1998; Golchin and Farahany, 2019).

Bone tissue engineering has emerged as an interdisciplinary strategy combining biomaterials, MSCs and/or biologically active molecules, aiming to reconstruct injured or lost bone (Place et al., 2009). Along with the direct relationship between osteoblastic lineage and bone formation, major developments were focused on osteoinductive materials able to induce MSCs osteoblastic differentiation, without chemical exogenous stimuli. Materials mimicking physicochemical and mechanical properties of bone extracellular matrix are developed to guide MSCs fate (Gao et al., 2017; Li et al., 2017; Zhang et al., 2018a). Indeed, MSCs sense physical and mechanical signals from their microenvironment and simultaneously convert them into environmental signals that regulate their behavior. We have recently developed a versatile osteoinductive coating made of organic chitosan/hyaluronic acid biopolymers and inorganic calcium phosphate, with a compositional analogy to human mineral bone and offers interesting properties for bone regenerative medicine, as it provides a suitable framework for MSCs osteogenic commitment (Rammal et al., 2017a).

MSCs are a heterogeneous population that contains a very low yield of cells able to differentiate into osteoblastic lineage. Once injected into a damaged tissue, MSCs showed a relatively poor rate of cell engraftment and engrafted ones are rather to be short-lived (Wang et al., 2014). Taken together, the current research seems to argue that MSCs differentiation contributes minimally to tissue regeneration while paracrine activities play a more predominant role. MSCs secrete cytokines, chemokines and growth factors to orchestrate tissue repair (i.e., by promoting angiogenesis and tissue regeneration and inhibiting fibrosis, apoptosis and inflammation) (Glenn and Whartenby, 2014; Wang et al., 2014; Haumer et al., 2018; Najar et al., 2018). Furthermore, an increase in bone resistance to fracture along with an increase in bone mineral density were reported following MSCs-based therapy for osteoporosis (Aghebati-Maleki et al., 2018; Saito et al., 2018). Local administrations of allogenic MSCs into the bone marrow cavity of irradiation-induced osteoporotic mice or of ovariectomy-induced osteoporotic rats were found to be effective against osteoporosis progression, to enhance bone apposition, and to promote freshly osteoid formation. Using MSCs to treat osteoporosis is already in clinical trials and no outcomes have been described (ClinicalTrials.gov identifier: NCT02566655; NCT01532076).

**Figure 1:**
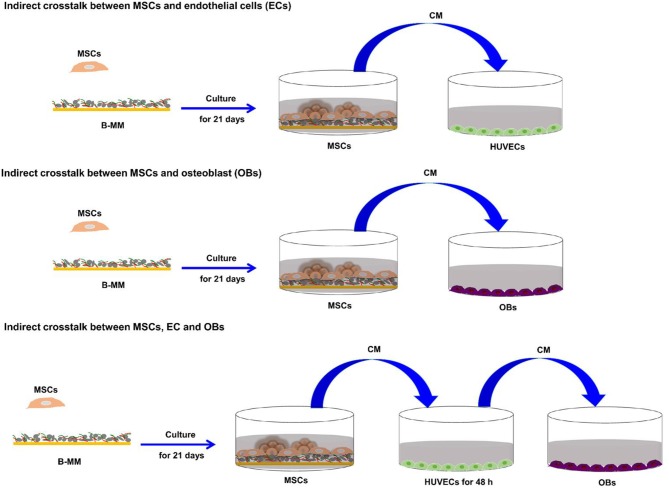
Representative schema of the study experimental design.

In light of these data, the present study investigates the capacity of bone-mimetic material (B-MM) to promote pro-regenerative secretome from MSCs especially on the production of either angiogenic or osteogenic factors (Figure 1). Our results provide, herein, evidences that the indirect crosstalk between MSCs and various cell types involved in bone regeneration, namely endothelial cells (ECs) and OBs, might be finely regulated by B-MM.

## Materials and Methods

### Experimental Design

To investigate the effect of bone-mimetic material (B-MM) on MSCs differentiation and secretome, Wharton's jelly derived MSCs were cultured for 21 days on both B-MM and glass. The study of the crosstalk between MSCs and EC or OBs was performed using MSCs culture media collected between 19th and 21st day of culture (Figure 1).

### Bone-Mimetic Material

#### Material

Calcium chloride hydrate (CaCl_2_, 2H_2_0: 0.32 M) and chitosan (low molecular weight: 0.3 mg/mL) were dissolved in NaCl (0.15 M)/HCl (2 mM) buffer pH 4 (A solution). Sodium dihydrogen phosphate hydrate (NaH_2_PO_4_: 0.19 M) and hyaluronic acid (molecular weight of 200 kDa: 0.3 mg/mL) were prepared in NaCl (0.15 M) buffer pH 10 (B solution). Both salt solutions were prepared in ultrapure water (Millipore®). Coverslips of 14 mm diameter were provided from Thermo Scientific. Each experiment was preceded by a cleaning step of the glass coverslips with sodium dodecyl sulfate (100 mM) for 15 min at 100°C. After an intensive ultrapure water (Millipore®) rinse, coverslips were brought in contact with HCl (100 mM) for 15 min at 100°C and finally rinsed with ultrapure water and kept at 4°C.

#### Substrate Build-Up

An automated spraying device was used for bone-mimetic material (B-MM) build-up. This device is constituted of four identical Airbrushes VL (Paasche®, USA) nozzles. Each nozzle is pressurized by in-house compressed air line under a pressure of 1 bar and connected to solenoid valves. The spraying of the different solutions, following a chosen deposition sequence, is obtained by a succession of closings and openings of the valves controlled by homemade software. Three nozzles allow spraying of the A solution, the B solution and of the rinsing solution. The fourth nozzle, free of solution, is used for the drying step. The cleaned coverslip is mounted vertically on a mobile holder. For homogenous B-MM build-up, the holder was rotated at 150 rpm. Both A and B solutions were sprayed simultaneously, on coverslip, for 2 s followed by a rinsing step of 2 s with ultrapure water and a drying step of 2 s under compressed air. These steps were repeated 50 times and polymer concentrations were adjusted after calculation of flows during spraying in order to keep a charge ratio hyaluronic acid/chitosan constant and equal to 0.7 to optimize the complex formation (Cado et al., 2012).

### Cell Culture

Human specimen (umbilical cord, mandibular bone specimen, and venous blood) harvestings were approved ethically and methodologically by our local Research Institution and were conducted with informed patients (written consent) in accordance with the usual ethical legal regulations (Article R 1243-57). All procedures were done in accordance with our authorization and registration number DC-2014-2262 given by the National “Cellule de Bioéthique.”

#### Wharton's Jelly Mesenchymal Stem Cells (MSCs)

MSCs were enzymatically isolated from fresh human umbilical cords obtained after full-term births (Mechiche Alami et al., 2014). MSCs were amplified at a density of 3 × 10^3^ cell/cm^2^ in α-MEM culture medium supplemented with 10% decomplemented fetal bovine serum (FBS), 1% Penicillin/Streptomycin/Amphotericin B and 1% Glutamax® (*v/v*, Gibco) and maintained in a humidified atmosphere of 5% CO_2_ at 37°C with a medium change every 2 days. At the fourth passage, MSCs were seeded in 24 well plates at 24 × 10^3^ cells/cm^2^ on UV-decontaminated B-MM or UV-decontaminated glass coverslip. MSCs were cultured for 21 days with a culture medium change every 2 days. Between 19th and 21st day of culture, MSCs culture media (MSCs-CM) were collected, centrifuged at 300 g and stored at −80°C. For a better comprehension, culture media from MSCs cultured on B-MM and glass were designated as CM_B−MM_ and CM_g_, respectively. Effective MSCs commitment into osteoblastic lineage was checked on cross sections of embedded paraffin samples according to previously published procedure (Mechiche Alami et al., 2017). Alizarin red staining was performed on consecutive tissue sections and images were taken using scanner iScan Coreo AU (Roche®, Ventana). For immunohistochemistry, after deparaffinization, 4 μm sections were incubated with the Cell Conditioner 1 (EDTA, pH 8.4) for 64 min, followed by preprimary peroxidase inhibition and incubation with the primary rabbit polyclonal antibody targeting osteocalcin (at a 1/100 dilution, Calbiochem) at 37°C overnight. Then, the staining reaction was performed using the UltraView Universal DAB v3 Kit (Ventana Medical System). Images were taken using scanner iScan Coreo AU.

#### Human Umbilical Cord Vein Endothelial Cells (HUVECs)

HUVECs were enzymatically isolated from fresh human umbilical cords veins obtained after full-term births following Jaffe et al., method (Rammal et al., 2017b). HUVECs were amplified at a density of 10^4^ cell/cm^2^ in endothelial basal medium (EBM)-2 supplemented with 20% decomplemented FBS, 1% Penicillin/Streptomycin/Amphotericin B and 1% Glutamax® (*v/v*, Gibco) and maintained in a humidified atmosphere of 5% CO_2_ at 37°C with a medium change every 2 days. At the second passage, HUVECs were cultured in 12 well plates at 10^4^ cells/cm^2^ in the presence of diluted CM_B−MM_, and CM_g_ (1:1 in EBM-2) for 48 h. HUVECs stimulated with recombinant human Tumor Necrosis Factor α (TNF α, R&D Systems) at 10 ng/mL for 48 h were used as inflammatory positive control. After 48 h, culture media were collected, centrifuged at 300 g and stored at −80°C. For a better comprehension, culture media of CM_B−MM_ and CM_g_ stimulated HUVECs were designated as EC-CM_B−MM_ and EC-CM_g_, respectively.

#### Human Mandibular Pre-osteoblasts (OBs)

Human mandibular bone specimens without any clinical or radiographic evidence of pathology were obtained from young patients (aged 13–33 years) undergoing windows teeth extraction oral surgery. After extensive (four to five times) washing steps, bone specimens were scraped to remove attached soft tissue and periosteum, broken into small pieces and predigested for 1 h with trypsin-EDTA (0.5%, *v/v*)/B collagenase (1 mg/mL) in a serum-free Dulbecco's Modified Eagle medium (DMEM). Fragments were then placed into 25 cm^2^ tissue culture flask and maintained in a humidified atmosphere of 5% CO_2_ at 37°C, allowing thus OBs migration and proliferation in the presence of DMEM supplemented with 20% FBS and 1% Penicillin/Streptomycin (*v/v*, Gibco). OBs were then amplified at a density of 10^4^ cell/cm^2^ in 10% FBS supplemented DMEM with a medium change twice/week and used at the third passage in our experimental study design. At the third passage, OBs were cultured in 24 well plates at 10^4^ cells/cm^2^ in the presence of diluted CM_B−MM_, CM_g_, EC-CM_B−MM_, and EC-CM_g_ (1:1 in DMEM) for 7 days. OB maintained in basal and osteogenic media (i.e., DMEM supplemented with 10 mM β-glycerophosphate, 250 μM L-ascorbic acid 2-phosphate and 5 nM dexamethasone) were used as controls.

#### Co-culture of Human Neutrophils With HUVECs

Neutrophils were purified from human whole blood collected on EDTA (BD Vacutainer® K2E, Franklin Lakes, USA) using the Polymorphprep™ protocol. Contaminating red blood cells were removed by a hypotonic shock. Resulting neutrophils were resuspended in complete RPMI 1640 media supplemented with 1% Penicillin/Streptomycin and 2.5% heat-inactivated autologous human serum and represented >97% of the cells. The PMNs were at least 95% viable. One million of neutrophils was finally brought in contact with un-stimulated and stimulated HUVECs (i.e., incubated for 48 h with CM_B−MM_ and CM_g_).

### ELISA Cytokines, Chemokines, and Growth Factors Release

Secreted levels, in MSCs culture media (CM_B−MM_ and CM_g_), of IL-1β, IL-6, IL-8, IL-10, transforming growth factor (TGF-β), osteoprotegerin (OPG), Prostaglandin E_2_ (PGE_2_), receptor activator of nuclear factor kappa-B ligand (RANKL), hepatocyte growth factor (HGF), vascular endothelial growth factor (VEGF), fibroblast growth factor (b-FGF), and bone morphogenic protein-2 (BMP-2) were assessed. ELISA MAX™ Deluxe kit for human IL-6, IL-8, IL-10, and b-FGF (BioLegend), DuoSet ELISA Kit for human IL-1β, TGF-β, Osteoprotegerin/TNFRSF11B, TRANCE/RANKL/TNFSF11, HGF, VEGF, and BMP-2 (R&D Systems, France) and PGE-2 ELISA Kit (Cayman Chemical) were used. Absorbance was measured according to the manufacturers' instructions.

### Transwell Migration Assay

HUVECs were seeded on the top of a cell culture insert membrane (Millicell® Hanging Cell Culture Inserts) at the density of 2 × 10^3^ cells/well. MSCs culture media (CM_B−MM_ and CM_g_) were deposited in the bottom of a 24 well plastic culture plate. After 48 h of incubation at 37°C in 5% CO_2_, non-migrating HUVECs were removed from the top of the membrane and migrated cells at the bottom of the insert membrane were fixed with methanol then stained with crystal violet. Migrated cells were finally imaged using EVOS® digital microscope and counted. EBM-2 and α-MEM ± 10% FBS were used as controls.

### PCR Gene Expression Analysis

For CM_B−MM_ and CM_g_ stimulated HUVECs and OBs as well as EC-CM_B−MM_ and EC-CM_g_ stimulated OBs (Figure 1, study experimental design), total RNA was isolated and purified using MasterPure™ RNA Purification Kit (Epicenter® Biotechnologies) in accordance with the manufacturer protocol. RNA purity was assessed by measuring the absorbance ratio at 260/280 nm (Nanodrop 2000C, ThermoScientific), which was comprised between 1.8 and 2. Total RNAs (500 ng) were reverse transcribed into cDNA using a High Capacity cDNA Reverse Transcription kit (Applied Biosystems) following manufacturer instructions. Ten nanograms of reverse transcription product were amplified by qRT-PCR on a StepOnePlus™ system (Applied Biosystems). Using this approach, the transcriptional levels of *RPS18* (internal control), *TNF*α, *IL-6, IL-8, SELE, ICAM1*, and *BMP-2* mRNA in stimulated HUVECs and the transcriptional levels of *HPRT-1* (internal control), *COL1A1*, and *BGLAP* mRNA in stimulated OBs were determined using Power SYBR® Green PCR Master MIX (Applied Biosystems) and TaqMan® Fast Advanced Master Mix (Applied Biosystems) for *ALPL* and *Runx2* mRNA. After a first denaturation step at 95°C for 10 min, qRT-PCR reactions were performed according to a thermal profile that corresponds to 40 cycles of denaturation at 95°C for 15 s, annealing and extension at 60°C for 1 min. Data analysis was performed with the StepOne™ Software v2.3 (Applied Biosystems).

### Scanning Electron Microscopy With a Field Emission Gun (FEG-SEM)

Neutrophils adhered to stimulated HUVECs (i.e., incubated for 48 h with CM_B−MM_ and CM_g_) were fixed with 2.5% (w/v) glutaraldehyde (Sigma Aldrich) at room temperature for 1 h. Samples were dehydrated in graded ethanol solutions from 50 to 100% and desiccated in hexamethyldisilazane (Sigma Aldrich) for 10 min. After air-drying at room temperature, samples were sputtered with a thin gold–palladium film under a JEOL ion sputter JFC 1100 and viewed using FEG-SEM (JEOL JSM-7900F). Images were acquired from secondary electrons at primary beam energy between 5 to 20 kV.

### Mitochondrial Activity

WST-1 cell proliferation assay (Roche Diagnostics) was performed on human mandibular osteoblasts (OBs) cultured in MSC (CM_B−MM_ and CM_g_) and stimulated HUVECs (EC-CM_B−MM_ and EC-CM_g_) culture media for 2, 4, and 7 days. Absorbance was measured at 440 nm using a FLUOstar Omega microplate reader (BMG Labtech) against a background control as blank. A wavelength of 750 nm was used as the correction wavelength. Mitochondrial activity, an indicator of cell viability, was calculated as the absorbance ratio between stimulated and basal culture medium (considered as 100% of viable osteoblasts).

### Statistical Analysis

All MSCs experiments were performed with six independent umbilical cords. HUVECs, OBs, and neutrophils, were performed with three independent donors. ELISA and PCR results are presented as box plot chart with median using GraphPad® Prism 5 software. Multivariate statistical analysis was performed by XL Stat software. Metabolic activity results are presented as histograms with mean ± standard error of the mean. All statistical analysis were performed using GraphPad® Prism 5 software. For Mann Whitney test, a value of *p* < 0.05 was accepted as statistically significant *p* (rejection level of the null-hypothesis of equal medians).

## Results and Discussion

**Figure 2:**
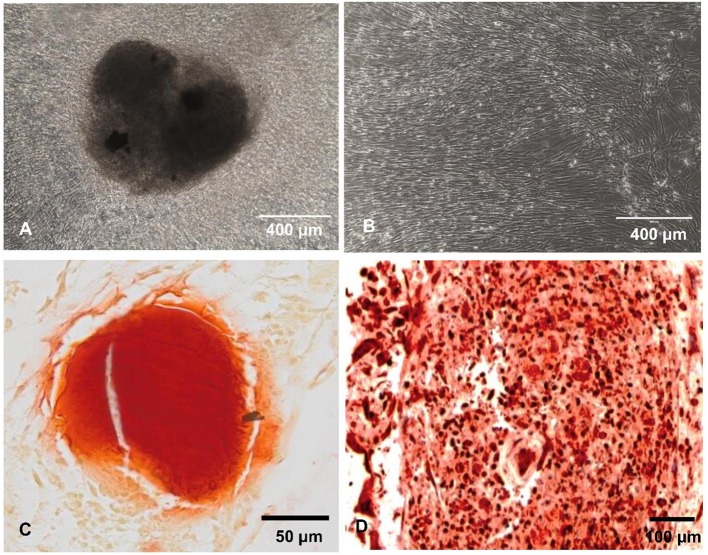
MSCs behavior. **(A,B)** Representative optical images showing MSCs accretions on bone-mimetic material **(A)** and cellular layer on glass **(B)** (scale bar 400 μm). **(C)** Red alizarin histological staining and **(D)** osteocalcin immunohistochemistry (scale bars 50 and 100 μm, respectively), demonstrating the bone*-like* nodule formation on B-MM.

**Figure 3:**
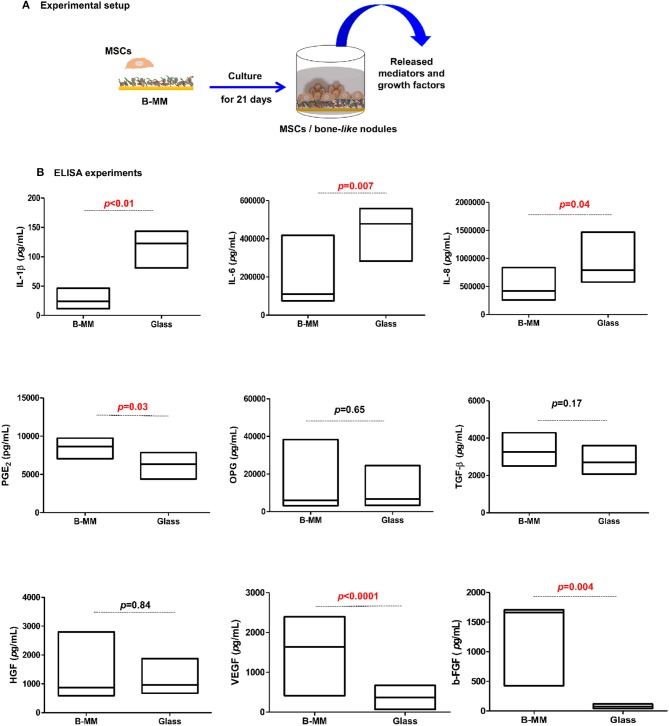
Cytokines, chemokines, and growth factors production. **(A)** Experimental setup and **(B)** Released IL-1β, IL-6, IL-8, PGE-2, OPG, TGF-β, HGF, VEGF, and b-FGF quantified by ELISA, indicating a down production of inflammatory mediators and up release of angiogenic mediators by MSCs cultured on bone-mimetic material (B-MM) compared to glass (*n* = 6, Mann Whitney test). TNF-α, IL-10, soluble RANKL, and BMP-2 were not detected in MSCs media.

**Figure 4:**
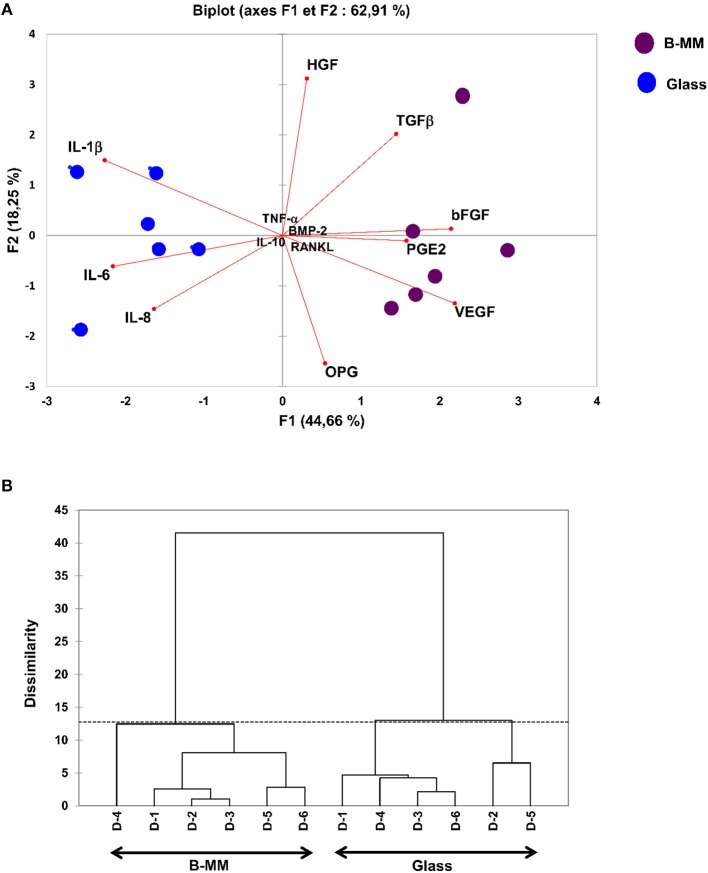
Multivariate statistical analysis. **(A)** Principal Component Analysis (PCA) plots and **(B)** Dendrogram, indicating, despite donor (D) variability, a clear separation between the averages of secreted mediators by MSCs cultured on bone-mimetic material (B-MM) and on glass.

We recently reported that intrinsic features of bone-mimetic material (B-MM) made from inorganic calcium phosphate supplemented with chitosan and hyaluronic acid biopolymers influences MSCs fate through mechanobiological pathway, inducing the expression of bone specific proteins (up to 1 week) (Rammal et al., 2017a). Bone develops through a tightly regulated process leading to a hierarchically ordered three-dimensional structure described in the literature as bone nodule (Mechiche Alami et al., 2016). Starting from day 14, MSCs cultured on B-MM formed 3D nodules in some distinct region (about 8% of the cultured area), whereas on control glass coverslip, no major morphological changes and no nodules were observed Histological and immunohistochemical analysis of paraffin-embedded nodules evidenced the presence of mineralized matrix positive to red alizarin and cells positive to osteocalcin (Figure 2 and Figure S1). A deeper characterization of these bone-*like* nodules would require multiscale investigations (Gentleman et al., 2009) that are out of scoop of the present study. The nodule density of around 9 ± 2 nodules in the cultured area suggests a low commitment of MSCs into osteoprogenitor cells. These observations are consistent with other studies highlighting a very low yield of MSCs able to differentiate into osteoblastic lineage (Jin and Lee, 2018; Golchin and Farahany, 2019). Along their capacity to differentiate into desired phenotype, MSCs contribute to tissue regeneration through the secretion of soluble and insoluble mediators (Glenn and Whartenby, 2014; Wang et al., 2014; Haumer et al., 2018; Najar et al., 2018). Focusing our investigations on the secretion of soluble mediators required for bone regeneration, the paracrine activity of MSCs cultured on B-MM was analyzed and compared to glass (Figure 3A, experimental design). ELISA results showed that MSCs cultured on B-MM decreased significantly the secretion of IL-1β, IL-6, and IL-8 pro-inflammatory mediators (≈ 5-, 4-, and 2-fold vs. glass, *p* < 0.01, *p* = 0.007 and *p* < 0.04, respectively, Mann Whitney test) but increased significantly PGE-2 production (≈ 1.37-fold vs. glass, *p* < 0.01, Mann Whitney test) (Figure 3B). TNF-α and RANKL were below the detection limit while the constitutive production of OPG was found to be not sensitive to culture substrate (Figure 3B). These mediators have great potential in bone repair and homeostasis (Raisz, 1999; Kon et al., 2001; Marsell and Einhorn, 2011; Sugimoto et al., 2016; Lin et al., 2018), but at high level, IL-1β, IL-6, IL-8, TNF-α, and soluble RANKL could be involved in osteoclast activation, bone destruction and ineffective regeneration (Mountziaris and Mikos, 2008). MSCs secrete various soluble growth factors to promote bone formation (TGF-β, VEGF and BMPs) and vascularization (HGF, b-FGF, VEGF) (Gerber et al., 1999; Tang et al., 2009; Chim et al., 2013; Crane et al., 2016). While the secretion of TGF-β and HGF was unchanged, the production of VEGF and b-FGF was significantly increased on B-MM (≈ 4.5- and 36-fold vs. glass, *p* < 0.001, Mann Whitney test). Surprisingly BMP-2 was not detected in MSCs supernatants (Figure 3B). Bone extracellular matrix (ECM) may not only sequester and store soluble BMPs but also expose them to OBs receptors; explaining away the lack of soluble BMP-2 detection in culture media (Chim et al., 2013). Despite its inability to induce MSCs osteogenic commitment, TGF-β promotes the recruitment and proliferation of osteoprogenitors during bone healing process (Chim et al., 2013). VEGF and b-FGF are known to promote ECs migration and tissue vascularization and indirectly OBs migration, proliferation, and differentiation (Gerber et al., 1999; Tang et al., 2009; Chim et al., 2013). Taken together, these results suggest that the paracrine activities of MSCs cultured on B-MM seems to be imbalanced in favor of vasculogenesis rather than osteogenesis. This conclusion was supported by principal component analysis (Wu et al., 2016) that showed data variance superior to 60% (Figure 4A). It appears that b-FGF, VEGF, HGF, TGF-β, OPG as well as PGE-2 are closer to MSCs on B-MM whereas IL-1β, IL-6, and IL-8 are closer to MSCs on glass. Discrimination of secretory activity was not affected by donor variability but resulting differences were due to MSCs behavior once on B-MM or glass substrate (Figure 4B).

### Indirect MSCs/Endothelial Cells (ECs) Crosstalk

**Figure 5:**
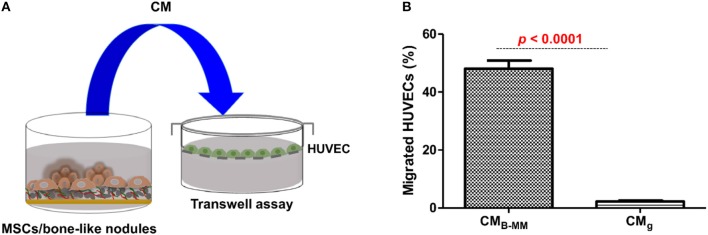
Endothelial cell recruitment. **(A)** Experimental design and **(B)** Transwell chemotaxis assay, showing a significant increase in endothelial cell migration in presence of MSCs culture media (MSCs-CM) in presence of bone-mimetic material (B-MM) compared to glass (for MSCs and HUVECs, *n* = 6 and 3, respectively, Mann Whitney test).

The crosstalk between MSCs/OBs/ECs is essential for bone formation and remodeling as well as around implanted graft during bone repair (Helmy et al., 2012). Above results suggest that MSCs cultured on B-MM could have a great potential in boosting ECs migration and proliferation (Gerber et al., 1999; Mountziaris and Mikos, 2008). To evaluate the effect of MSCs cultured on B-MM on ECs migration, transwell chemotaxis assay was performed. MSCs culture media (MSCs-CM) harvested between 19th and 21st day of culture and primary HUVECs were used (Figure 5A, experimental design). For better comprehension, we designated culture media from MSCs cultured on B-MM and on glass by CM_B−MM_ and CM_g_, respectively. CM_B−MM_ enhanced significantly HUVECs recruitment with about 50% of migrated HUVECs whereas CM_g_ recruited only 2% (Figure 5B), confirming the superior chemotactic activity of MSCs on B-MM compared to glass.

**Figure 6:**
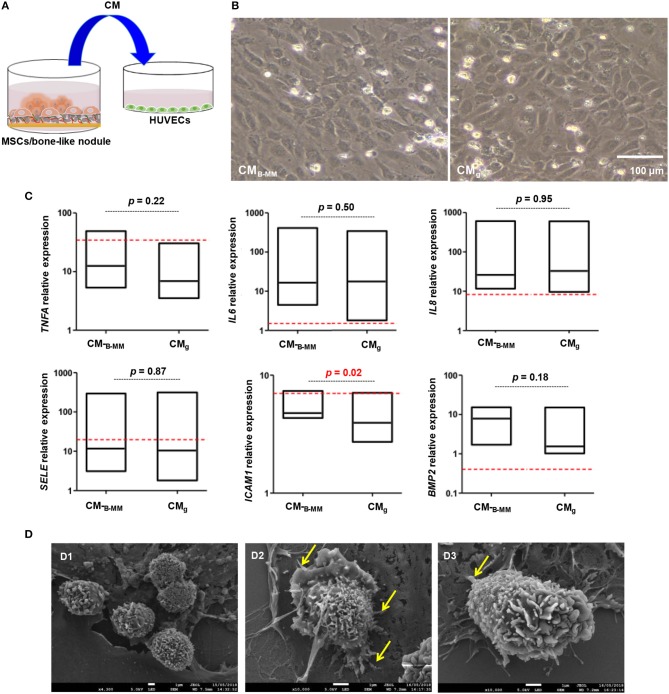
Endothelial cell inflammatory phenotype. **(A)** Experimental design and **(B)** representative optical images of stimulated HUVECs by MSCs culture media (MSCs-CM_B−MM_ and MSCs-CM_g_, scale bar = 100 μm), highlighting a cobblestone morphology. **(C)** Gene expression of *ICAM1, SELE, TNFA, IL6, IL8*, and *BMP2* quantified by qRT-PCR (red bars indicate TNF-α stimulated endothelial cells positive control) (for MSCs and HUVECs, *n* = 6 and 3, respectively, Mann Whitney test), showing a significant over-expression of *ICAM1* in presence of CM_B−MM_. **(D)** Endothelial cell/neutrophil interaction imaged by scanning electron microscopy (Yellow arrows indicating neutrophil protrusion, scale bars = 1 μm), showing spread neutrophils with a transmigrating aspect in presence of CM_B−MM_.

Upon bone graft implantation, recruited leukocytes secrete an array of pro-inflammatory cytokines and growth factors, orchestrating the graft integration into host bone tissue (Luu et al., 2013). ECs through the expression of adhesion molecules and the release of inflammatory cytokines, constitute the main regulators of leukocyte recruitment (Ucuzian and Greisler, 2007; McGettrick et al., 2012). In contrast to differentiated MSCs, naïve cells, mainly through IL-6 and TGF-β release, are known to regulate leukocyte diapedesis (Ucuzian and Greisler, 2007). Although B-MM slightly increased TGF-β release, above cited results indicated a significant decrease in IL-6 production (Figure 3B), suggesting a potential indirect effect on leukocyte recruitment. Thus, a second set of experiments was conducted to investigate the latter hypothesis. HUVECs were incubated with CM_B−MM_ and CM_g_ for 48 h and qRT-PCR experiments were conducted to assess HUVECs inflammatory phenotype through gene regulation of *TNFA, IL6, IL8, SELE*, and *ICAM1* (Figure 6A, experimental design). Note that once stimulated, ECs kept their characteristic cobblestone morphology (Figure 6B). Compared to un-stimulated HUVECs (i.e., cultured in endothelial basal medium), qRT-PCR results revealed an up-regulation of all studied genes in CM_B−MM_ and CM_g_ stimulated HUVECs (at least an up-regulation of 2^−Δ*ΔCT*^), signature of inflammatory phenotype. Regardless ECs inflammatory phenotype and compared to TNF-α stimulus (positive inflammatory control), MSCs-CM had a lower inflammatory impact on *ICAM1, SELE*, and *TNFA*, but up-regulated both *IL6* and *IL8*. Interestingly, although no differences were observed for *TNFA, IL6, IL8*, and *SELE*, we noticed a significant over-expression of *ICAM1* in presence of CM_B−MM_ (1.5-fold vs. CM_g_, *p* < 0.02, Mann Whitney test, Figure 6C).

ICAM-1, constitutively expressed at low levels on ECs, can be up-regulated in response to pro-inflammatory stimuli, mediating neutrophil diapedesis (Anderson et al., 2008). Thus, adhesion of neutrophils to CM_B−MM_ and CM_g_ stimulated HUVECs was followed. Whatever the studied condition, the cobblestone monolayer integrity was altered when neutrophils were added. SEM revealed that neutrophils, in close similarity with TNF-α stimulated HUVECs condition, exhibited membrane ruffling and vesicles, indicative of neutrophil activation in contact with CM_B−MM_ stimulated HUVECs; whereas in contact with CM_g_ stimulated HUVECs, neutrophils, as for un-stimulated HUVECs condition, were rounded and appeared less activated (Figure 6D and Figure S2). Furthermore, a higher magnification of activated neutrophils in close contact to the apical part of HUVECs showed elongated neutrophils with long protrusions embracing HUVECs and forming adhesion points (Figure 6D, arrows). These observations corroborate previous studies, indicating that spreading can be signature of transmigrating neutrophils (Anderson et al., 2008; Schaefer et al., 2014).

Adding ECs to bone engineered constructs increases vascularization within and surrounding implanted constructs and boosts bone formation (Gerber et al., 1999; Von Wedel-Parlow et al., 2011). In response to VEGF, ECs produce BMP-2 that stimulates OBs differentiation, promoting fracture healing (Zhang et al., 2018b). Regarding the significant increase in VEGF release by MSCs cultured on B-MM vs. glass (Figure 3B); the expression of BMP-2 by HUVECs was followed. Despite a significant *BMP2* up-regulation in CM_B−MM_ stimulated HUVECs (≈ 5^−Δ*ΔCT*^, Figure 6C), released BMP-2 was not detected in the culture supernatant, suggesting that BMP-2 production is under the detection threshold of the kit or is accumulated within the cell cytoplasm. These results are consistent with other observations demonstrating that ECs require direct contact with bone marrow derived MSCs for the effective production of BMP-2 (Kaigler et al., 2005).

### Do MSCs Cultured on B-MM Promote Indirectly Pre-osteoblasts Differentiation?

**Figure 7:**
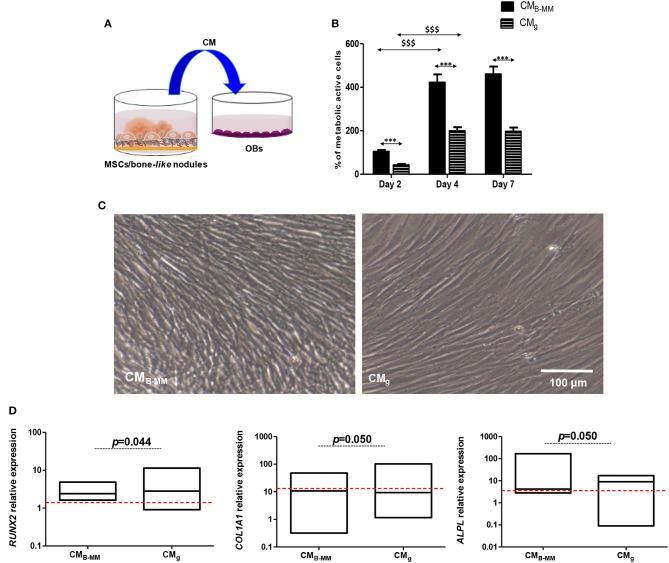
Indirect MSCs/pre-osteoblasts crosstalk. **(A)** Experimental design and **(B)** pre-osteoblast (OBs) proliferation in presence of MSCs culture media (MSCs-CM). Results normalized to un-stimulated OBs, showing a significant increase in OB proliferation in presence of MSCs-CM cultured on bone-mimetic material compared to inert glass (*CM_B−MM_ vs. CM_g_ and ^$^D4 and D7 vs. D2, for MSCs and OBs, *n* = 6 and 3, respectively, Mann Whitney test). **(C)** Optical images of stimulated OBs after 7 days of culture in MSCs-CM_B−MM_ and MSCs-CM_g_, indicating fibroblastic cell morphology (Scale bars = 100 μm). **(D)** Gene expression of *RUNX2, COL1A1*, and *ALPL* quantified by qRT-PCR (red bars indicates OBs cultured in presence of osteogenic media), indicating the absence of an effective osteoblastic differentiation in the studied conditions (for MSCs and OBs, *n* = 6 and 3, respectively, Mann Whitney test). *** or $$$ means *p* < 0.0001. * is media dependent for the same kinetic time point and the $ is kinetic time point dependent for the same media.

As shown above, IL-1β, IL-6, IL-8, and VEGF secretion levels from MSCs cultured on B-MM were significantly modulated compared to glass (Figure 3B). These soluble factors are reported to mediate, in dose-dependent manner, pre-osteoblasts proliferation and differentiation (Mountziaris and Mikos, 2008). In the following, proliferation kinetic of human alveolar bone derived OBs cultured in presence of CM_B−MM_ and CM_g_ was firstly investigated (Figure 7A, experimental design). When cultured in CM_B−MM_, the proliferation of OBs was significantly promoted compared to OBs cultured in osteogenic media (Figure S3). Moreover, we noticed a significant increase in MSCs-CM stimulated OBs proliferation from day 2 to 4 with a slow proliferation rate for CM_g_ (*p* < 0.0001, Mann Whitney test). Whatever the stimulus, a plateau appeared from day 4 to 7 (Figure 7B). No significant difference in OBs morphology was noticed among the studied conditions (Figure 7C), suggesting that MSCs-CM stimuli did not affect the cell cytoskeleton. Differentiation of OBs cultured in presence of MSCs-CM was secondly investigated. During the early stage of bone formation, the gene expression of Runx-2 and ECM proteins including COL-I is concomitant with OBs proliferation whereas the non-proliferative and immature OBs up-regulate certain genes such as alkaline phosphatase (*ALPL*), a specific enzyme involved in the mineralization process. The late stage of bone formation corresponds to matrix mineralization, which is characterized by an up-regulation of non-collagenous proteins such as osteocalcin (*BGLAP*) (Mechiche Alami et al., 2017). While MSCs-CM stimulated the OBs proliferation, we failed to observe their effective differentiation (Figure 7D). Indeed, as for OBs cultured in osteogenic media, qRT-PCR experiments showed that both MSCs-CM up-regulate the expression levels of *RUNX2, COL1A1*, and *ALPL* over the time, however no statistically significance was distinguished between the studied conditions. Furthermore, no boosting effect of OBs differentiation was observed as *BGLAP*, late bone specific marker, was not detected.

### Do MSC-CM Stimulated HUVECs Boosts Pre-osteoblasts Differentiation?

**Figure 8:**
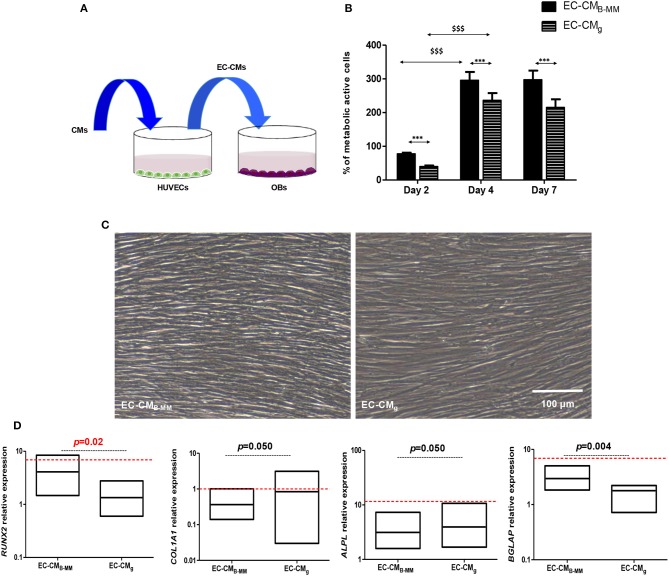
Indirect MSCs/HUVECs/pre-osteoblasts crosstalk. **(A)** Experimental design and **(B)** pre-osteoblast (OBs) proliferation in presence of endothelial cell-culture media (ECs-CM). Results normalized to un-stimulated OBs, showing a significant increase in OBs proliferation in presence of EC-CM (HUVECs stimulated with CM of MSCs cultured on bone-mimetic material and glass, *EC-CM_B−MM_ vs. EC-CM_g_ and ^$^D4 and D7 vs. D2, for MSCs and OBs, *n* = 6 and 3, respectively, Mann Whitney test). **(C)** Optical images of stimulated OBs after 7 days of culture in EC-CM_B−MM_ and EC-CM_g_, indicating fibroblastic cell morphology (Scale bars = 100 μm). **(D)** Gene expression of *RUNX2, COL1A1, ALPL*, and *BGLAP* quantified by q-RT-PCR (red bars indicates OBs cultured in presence of osteoinductive medium), suggesting an acceleration of the osteoblastic differentiation in presence with EC-CM_B−MM_ (for MSCs and OBs, *n* = 6 and 3, respectively, Mann Whitney test). *** or $$$ means *p* < 0.0001. * is media dependent for the same kinetic time point and the $ is kinetic time point dependent for the same media.

The intimate association between ECs and OBs suggests that ECs are to be prime sources for bone development (Gerber et al., 1999). In this part of the study, we assessed the effect of stimulated HUVEC supernatant (ECs-CM) on OBs (Figure 8A). As noticed above, we had a significant increase in OBs proliferation from day 2 to 4 with plateau from day 4 to 7 without changes in cell morphology (Figures 8B,C). However, proliferation rate of OBs seemed slower in presence of ECs-CM compared to MSCs-CM (≈ 300 vs. 450% after 4 days of culture). Investigating the OBs differentiation in presence of ECs-CM, qRT-PCR experiments showed a down regulation of *COL1A1* and *ALPL* concomitantly to an up-regulation of *RUNX2* and *BGLAP* after 7 days (Figure 8D). Thus, despite the absence of BMP-2 release by HUVECs, compared to CM_g_, CM_B−MM_ stimulated ECs up-regulated significantly *RUNX2* and *BGLAP* in OBs, suggesting a potential acceleration of OB differentiation.

## Conclusion and Perspectives

Herein we show that MSCs cultured on osteoinductive B-MM formed bone*-like* nodules. These nodules, arising from MSCs fibroblastic layers, occupied only 8% of cultured area. Despite the small fraction of committed osteoprogenitors, B-MM decreased the production of IL-1β, IL-6, and IL-8 inflammatory mediators and increased the release of b-FGF, VEGF angiogenic growth factors. Compared to CM_g_, CM_B−MM_ enhanced endothelial cell migration and neutrophil diapedesis via *ICAM1* up-regulation. Regarding osteogenesic capacities, both CM_B−MM_ and CM_g_ failed to boost pre-osteoblast differentiation. Interestingly, EC-CM_B−MM_ up-regulated BGLAP expression, suggesting an acceleration of pre-osteoblast maturation. Our results showed that BMP-2 seemed not responsible for ECs osteogenic property, suggesting the implication of other mediators. A deeper characterization (i.e., microarray analysis) of collected media is required to highlight the implication of other factors. MSCs and ECs are known to release therapeutic microvesicles that can act in a paracrine manner on tissue healing (Kaigler et al., 2005; Behera and Tyagi, 2018). Thus, it will be interesting to investigate the potential contribution of B-MM on microvesicles production and features.

## Data Availability Statement

All data generated or analyzed during this study are included in this published article (and its Supplementary Information Files). If not, they are available from the corresponding author on reasonable request.

## Ethics Statement

The studies involving human participants were reviewed and approved by our local Research Institution and were conducted with informed patients (written consent) in accordance with the usual ethical legal regulations (Article R 1243-57). All procedures were done in accordance with our authorization and registration number DC-2014-2262 given by the National Cellule de Bioéthique. The patients/participants provided their written informed consent to participate in this study.

## Author Contributions

HR, LE, MD, NB, AM, and JS: participated in experiments designing and performance. HR: materials conception and cell culture. LE: qRT-PCR. MD: ELISA experiments. NB: scanning electron microscopy. JS: technical support. AM: neutrophils co-culture assay. HR, CM, SG, and HK: participated in study design and manuscript writing. All authors reviewed the results and approved the final version of the manuscript.

### Conflict of Interest

The authors declare that the research was conducted in the absence of any commercial or financial relationships that could be construed as a potential conflict of interest.
