# Supplementary material for: Osteoinductive Material to Fine-Tune Paracrine Crosstalk of Mesenchymal Stem Cells With Endothelial Cells and Osteoblasts
Source: Front Bioeng Biotechnol. 2019 Oct 9;7:256. doi: 10.3389/fbioe.2019.00256 (PMC6795130; doi:10.3389/fbioe.2019.00256)
Supplement: Supplementary file 1 [file Data_Sheet_1.pdf]

Supporting Information for publication

Osteoinductive material to fine-tune paracrine crosstalk of mesenchymal stem cells with endothelial cells and osteoblasts

Hassan Rammal[1,2], Laura Entz[1], Marie Dubus[1,2], Aurélie Moniot[1], Nicolae B. Bercu[3], Johan Sergheraert[1,2,4], Sophie C. Gangloff[1,5], Cédric Mauprivez[1,2,4], Halima Kerdjoudj[1,2].*

1. EA 4691, Biomatériaux et Inflammation en Site Osseux (BIOS), SFR CAP Santé (FED4231), Université de Reims Champagne Ardenne, Reims, France.

2. UFR d'Odontologie, Université de Reims Champagne Ardenne, Reims, France.

3. EA 4682, Laboratoire de Recherche en Nanoscience (LRN), Université de Reims Champagne-Ardenne, Reims 51100, France.

4. Pôle Médecine bucco-dentaire, Hôpital Maison Blanche, Centre Hospitalier Universitaire de Reims, France.

5. UFR de Pharmacie, Université de Reims Champagne Ardenne, Reims, France.

Correspondence:

Dr. Halima KERDJOUDJ. EA 4691 « Biomatériaux et Inflammation en Site Osseux », Pôle Santé, UFR d'Odontologie, SFR-CAP Santé (FED 4231), URCA, 1 Avenue du Maréchal Juin, 51100 Reims, France.

E-mail: halima.kerdjoudj@univ-reims.fr.

Phone: (+33)3 26 91 80 12

Fax: (+33)3 26 91 80 12

Supporting Information for publication

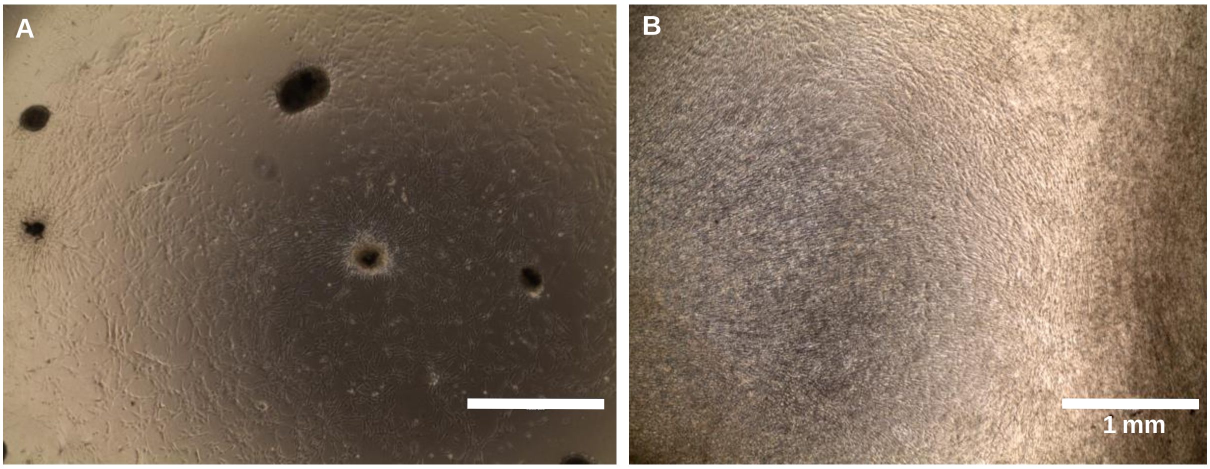

Figure S1: *MSCs behaviour*. A and B: Representative optical images showing MSCs accretions on bone-mimetic material (A) and cellular layer on glass (B) (scale bar 1 mm).

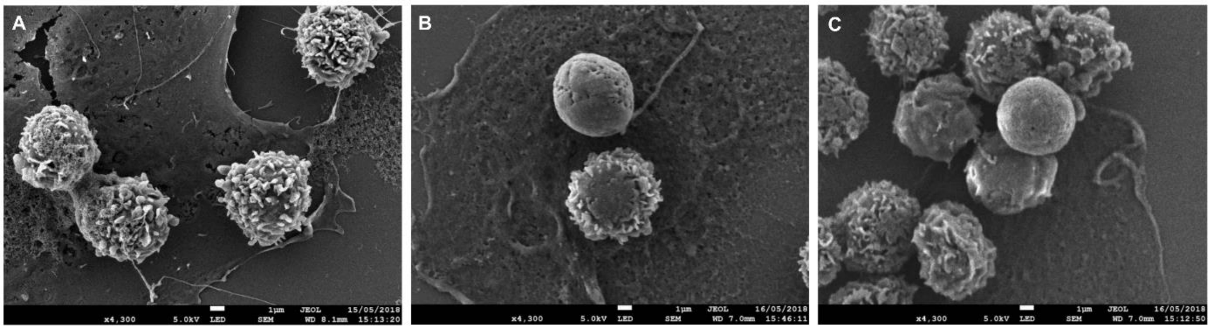

Figure S2: *Endothelial cell inflammatory phenotype.* Endothelial cell/neutrophil interaction imaged by scanning electron microscopy (scale bars = 1 µm), showing rounded and less activated neutrophils in contact of CM_g stimulated (A) and unstimulated HUVECs (B), and elongated and activated neutrophils in contact with TNF-α stimulated HUVECs (C).

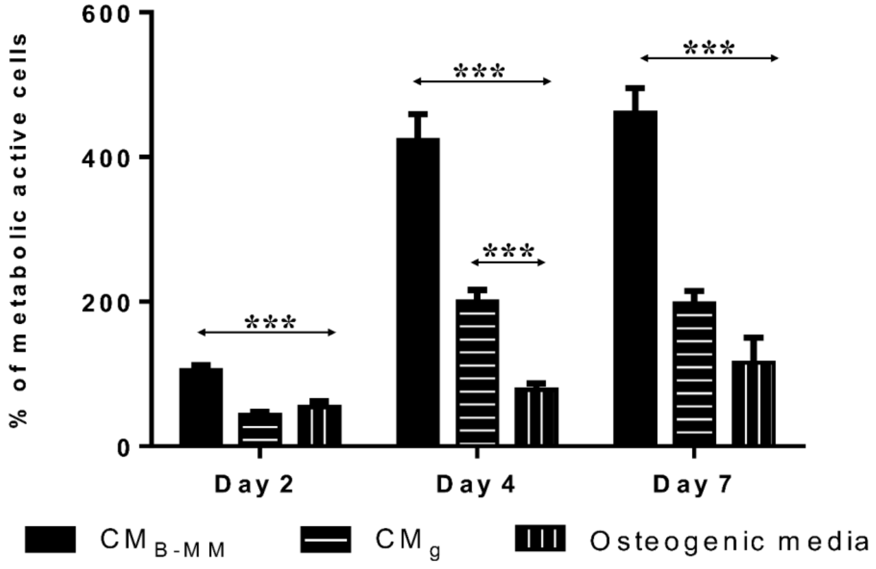

Figure S3: *Indirect MSCs/pre-osteoblasts crosstalk*. Pre-osteoblast (OBs) proliferation in presence of MSCs conditioned media (MSCs-CM) and osteogenic media. Results normalized to un-stimulated OBs, showing a significant increase in OB proliferation in presence of MSCs-CM cultured on bone-mimetic material compared to osteogenic media and inert glass. (MSCs and OBs, *n* = *6* and = *3*, respectively, Mann Whitney test).
